# Biomarkers of apoptosis and survival in esophageal squamous cell carcinoma

**DOI:** 10.1186/1471-2407-9-310

**Published:** 2009-09-03

**Authors:** Mikiko Takikita, Nan Hu, Jian-zhong Shou, Quan-Hong Wang, Carol Giffen, Philip R Taylor, Stephen M Hewitt

**Affiliations:** 1 Tissue Array Research Program, Laboratory of Pathology, Center for Cancer Research, National Cancer Institute, National Institutes of Health, Bethesda, MD, USA; 2 Genetic Epidemiology Branch, Division of Cancer Epidemiology and Genetics, National Cancer Institute, National Institutes of Health, Bethesda, MD, USA; 3 Cancer Institute and Hospital, Chinese Academy of Medical Sciences, Beijing, PR China; 4 Shanxi Cancer Hospital, Taiyuan, Shanxi, PR China; 5 Information Management Services, Inc, Silver Spring, MD, USA

## Abstract

**Background:**

Cancer of the esophagus is a deadly malignancy, and development of biomarkers that predict survival is an urgent need. The apoptotic pathways have been hypothesized as important in progression of esophageal squamous cell carcinoma (ESCC). We investigated a panel of proteins that regulate apoptosis as candidate of biomarkers of prognosis in ESCC.

**Methods:**

Tissue microarray (TMA) including 313 surgically-resected cases of ESCC specimens was built for immunohistochemical interrogation. We evaluated seven genes in the FasL-Fas apoptotic pathway - FasL, Fas, FAS-associated death domain protein (FADD), phosphorylated-FADD, and caspase 8 and 10, and the antiapoptotic protein bcl-2. We studied pathway integrity and relations to risk and clinical factors, and determined the prognostic significance of each marker.

**Results:**

Five markers showed strong inter-marker correlations (r ≥ 0.28, p < 0.001), including FasL, Fas, FADD, and caspases 8 and 10. FasL and FADD also showed modest correlations with one or more cancer risk factors, but none of the markers was significantly associated with either tumor stage or lymph node metastasis, the only two clinical factors that predicted survival in these ESCC cases. Multivariate-adjusted proportional hazard regression models showed no association between protein expression and risk of death for any of the seven markers examined.

**Conclusion:**

Individual biomarkers in the apoptosis pathway do not appear to predict survival of patients with ESCC.

## Background

Fas-mediated apoptosis is thought to be involved in the initiation and development of esophageal squamous cell carcinoma (ESCC). Previous gene expression profiling of ESCC showed over-expression of FAS-associated death domain RNA (*FADD*) and under-expression of *Fas *and *caspase 8 *[1]. The phosphorylated form of FADD (p-FADD) has recently been reported to regulate apoptotic activity [2]. Although the role of p-FADD in ESCC outcome is unclear, higher levels of p-FADD protein correlated with reduced survival in patients with lung adenocarcinomas [3] and prostate cancer [4].

Using an ESCC tissue microarray (TMA) [5], we explored the expression of FasL, Fas, FADD, p-FADD, caspase 8 and 10, which are proteins involved in the FasL-Fas apoptotic pathway, and the antiapoptotic protein bcl-2. We determined the prevalence of protein expression for each marker, investigated pathway integrity by evaluating the correlations between individual markers as well as between markers and risk factors/clinico-pathologic features, and we examined the prognostic significance of the markers on the survival of ESCC cases.

## Methods

### Patient selection

This study was approved by the Institutional Review Boards of the Shanxi Cancer Hospital and the U.S. National Cancer Institute. Patients presenting to the Shanxi Cancer Hospital in Taiyuan, Shanxi, People's Republic of China between 1996 and 2001 were eligible for inclusion in this study. The Shanxi Cancer Hospital, the largest cancer hospital in Shanxi, performed surgery on approximately 2000 new esophageal annually during the study period. We included cases in this study who: (i) were males or females 20 years of age or older, (ii) had newly diagnosed (incident) cancer of the esophagus without previous treatment (including surgery, chemotherapy, or radiotherapy), (iii) underwent surgical resection of their tumor at the Shanxi Cancer Hospital, and (iv) had their diagnosis histologically confirmed. Since a primary objective of this study was to evaluate somatic changes in tumors, we limited recruitment to patients who had complete surgical resection of their tumor as their primary therapy; approximately 50% of new ESCC cases underwent surgical resection as their primary therapy. Neoadjuvant and adjuvant therapy were not employed at the Shanxi Cancer Hospital in surgically resected ESCC cases during the time period that this study was conducted. Esophageal cancer cases were limited to those with histological ESCC, which included nearly all esophageal cancers since adenocarcinoma of the esophagus is essentially nonexistent in this high-risk population. All histological diagnoses were made initially by pathologists at the Shanxi Cancer Hospital and confirmed by pathologists at the National Cancer Institute. In addition to confirmation of their histologic diagnosis, cases were classified as either well differentiated or poorly differentiated ESCC.

We collected information on demographic and lifestyle cancer risk factors [eg, smoking, alcohol drinking, family history of upper gastrointestinal (UGI) cancer] on cases using a structured interview with a questionnaire administered by a nurse in the hospital prior to surgery. Clinical data was abstracted from hospital records after surgery. Demographic, lifestyle and clinical data for cases included in this study are shown in Table 1. All patients (or their family members) were re-contacted in 2003 to ascertain vital status.

**Table 1:** Characteristics of patients in apoptosis biomarker protein expression tissue microarray study

Risk factor	Prevalence of risk factor or clinicopathologic feature (N = 265)
Gender (male)	0.66
Age (years, median)	58
Tobacco use (yes)	0.60
Alcohol use (daily or weekly)	0.22
Family history of upper gastrointestinal cancer (yes)	0.27
	
**Clinico-pathologic feature**	
Tumor grade	
I	0.17
II	0.60
III	0.23
IV	0.004
Tumor stage	
1	0.004
2	0.13
3	0.86
4	0.01
Lymph node metastasis (yes)	0.45
Degree differentiation (poor)	0.47

### Tissue microarray (TMA) construction

Details of the TMA construction were previously described [5]. In brief, the TMA was constructed with surgical resection tissue samples from 313 ESCC cases, and selected control tissues using 0.6 mm needles. After exclusion of cores with inadequate tissue following sectioning and tissue transfer, the final immunohistochemical analyses included cores from 265 ESCC cases. Each of the 265 different ESCC cases contributed to one or more of the different biomarker analyses. Final numbers of cases for each of the biomarkers evaluated here are shown in Table 2.

**Table 2:** Distribution of protein expression of the apoptotic and antiapoptotic markers, Cox proportional hazard ratios (HR) and 95% confidence intervals (CI) for overall survival by the biomarker status for ESCC patients.

Gene protein	N^1^	Distribution of protein expression by overall score (N, prevalence)^2^									HR^3^ (95% CI) *P*-value	Number (prevalence) with positive protein expression score^4^	HR^5^ (95% CI) *P*-value
		Score											
		0	1	2	3	4	6	8	9	12			
**FasL**	244	15 (0.06)	48 (0.20)	92 (0.38)	80 (0.33)	3 (0.01)	6 (0.03)	---	---	---	1.10 (0.95-1.27) 0.45	9 (0.04)	1.04 (0.45-2.40) 0.92
**Fas**	244	24 (0.10)	49 (0.20)	44 (0.18)	91 (0.37)	8 (0.03)	22 (0.09)	---	5 (0.02)	1 (0.00)	0.95 (0.87-1.04) 0.07	36 (0.15)	0.79 (0.49-1.27) 0.32
**FADD**	248	10 (0.04)	122 (0.49)	72 (0.29)	8 (0.03)	17 (0.07)	12 (0.05)	4 (0.02)	1 (0.00)	2 (0.01)	1.05 (0.96-1.14) 0.79	36 (0.15)	1.22 (0.77-1.92) 0.39
**pFADD**	247	190 (0.77)	48 (0.19)	9 (0.04)	---	---	---	---	---	---	1.02 (0.76-1.38) 0.25	0 (0.00)	---
**Caspase 8**	244	11 (0.05)	76 (0.31)	144 (0.59)	---	11 (0.05)	2 (0.01)	---	---	---	0.92 (0.76-1.11) 0.53	13 (0.05)	1.03 (0.51-2.05) 0.94
**Caspase 10**	235	6 (0.03)	47 (0.20)	161 (0.69)	---	17 (0.07)	3 (0.01)	---	1 (0.00)	---	1.07 (0.93-1.23) 0.46	21 (0.09)	1.36 (0.81-2.27) 0.25
**Bcl2**	253	23 (0.09)	167 (0.66)	58 (0.23)	---	5 (0.02)	---	---	---	---	1.01 (0.81-1.27) 0.63	5 (0.02)	1.29 (0.52-3.25) 0.58

^1^N shown is for each individual biomarker and is less than 265 total because none of 7 biomarkers had data for all 265 ESCC cases.

^2^Overall score calculated as (intensity score) times (percent cells positive score) as described in methods.

^3^HR = hazard ratio (95% confidence interval) for increase in score of one category; from multivariate Cox proportional hazards model, adjusted for gender, age, tobacco use, alcohol use, family history of UGI cancer, tumor grade, tumor stage, metastasis, and degree differentiation.

^4^Positive protein expression = overall score ≥ 4 (negative = overall score ≤ 3).

^5^HR = hazard ratio (95% confidence interval) for positive protein expression (versus negative); from multivariate Cox proportional hazards model, adjusted for gender, age, tobacco use, alcohol use, family history of UGI cancer, tumor grade, tumor stage, metastasis, and degree differentiation.

### Immunohistochemistry staining and assessment

The TMA sections were stained with antibodies to FasL (Lab Vision Corp., CA), Fas (Santa Cruz, CA), FADD (Novocastra, UK), p-FADD (Cell Signaling Technology, MA), caspase 8 (Lab Vision Corp., CA), caspase 10 (Cell Signaling Technology, MA), and bcl-2 (Dako, CA). Slides were stained according to manufacturer's protocols. The immunohistochemical staining patterns were validated against previously described patterns of staining for each marker on a separate TMA, including appropriate positive and negative controls. Internal positive and negative controls, including normal squamous epithelium of the esophagus from non-cancer patients were utilized as available to further support the staining patterns.

Fas and FasL was expressed exclusively in cell membranes, whereas immunoreactivity of FADD and bcl-2 was cytoplasmic. Caspase 8 and 10 expression was detected in both cytoplasm and nucleus. p-FADD was primarily expressed in the nucleus of cells. Staining results were scored based on: (i) percent of positive tumor cells in tumor tissue: zero (0%), 1 (1-25%), 2 (26-50%), 3 (51-75%) and 4 (76-100%); and (ii) signal intensity: zero (no signal), 1 (weak), 2 (moderate) and 3 (marked). An overall score was calculated by multiplying the percent cells positive score by the intensity score (range zero to 12).

### Statistical analysis

All statistical analyses were performed using Statistical Analysis Systems (SAS) (SAS Corp, NC). Spearman correlation coefficients were used to assess associations between the seven different apoptosis biomarkers, and between the seven markers and risk factors/clinico-pathologic features. Overall survival time was calculated as the date of surgery to the date of death or the date last known alive. Survival was examined graphically with Kaplan-Meier curves and analyzed statistically with log-rank tests and proportional hazards regression models (SAS PHREG procedure) adjusted for lifestyle and tumor characteristics as covariates as previously described [6]. All *P*-values were two-sided and considered statistically significant if *P *< 0.05.

## Results

Overall protein expression for the seven markers evaluated here showed low levels of positivity, ranging from zero to 15% positive (Table 2). The Spearman correlation coefficients revealed that expression of FasL and Fas, FasL and caspase 10, FADD and caspase 8, FADD and caspase 10, and caspases 8 and 10 in ESCC were strongly associated (r ≥ 0.28, p < 0.0001). Fas and caspase 8, and Fas and caspase 10 were also significantly correlated (r = 0.18, p < 0.01). Moreover, FasL and FADD, FasL and caspase 8, and caspase 10 and bcl-2 were mildly associated (r = 0.15 to 0.16, p < 0.05) (Table 3).

**Table 3:** Spearman correlations between 7 apoptosis biomarkers^1^

	FasL	Fas	FADD	pFADD	Caspase8	Caspase10	Bcl2
**FasL**	1.00	0.30***	0.16*	-0.05	0.15*	0.29***	0.07
**Fas**		1.00	0.10	-0.04	0.18**	0.18**	0.00
**FADD**			1.00	-0.05	0.28***	0.29***	0.08
**phospho-FADD**				1.00	0.01	-0.04	0.03
**Caspase8**					1.00	0.29***	0.03
**Caspase10**						1.00	0.15*

**Bcl2**							1.00

^1^N varies from 231 to 244 and is less than 265 total because none of 7 biomarkers had data for all 265 ESCC cases

* p < 0.05

** p < 0.01

*** p < 0.0001

We successfully contacted 261 of 265 ESCC cases or their families during follow-up for vital status; 181 cases died and 80 were still alive at the end of follow-up. Median overall survival was 677 days (one year, 10 months) and 15 cases were still alive five or more years post-surgery; the longest survivor was still alive six years and two months after surgery. Kaplan-Meier graphs and log-rank analyses of individual markers did not reveal differences in overall survival by protein expression positivity (data not shown). Analyses adjusted for demographic attributes and potential confounding factors in Cox proportional hazard regression models also failed to identify significant associations between markers and survival time (Table 2).

Table 4 shows the Spearman correlations between the seven apoptosis biomarkers and five risk factors, which include sex, age, tobacco use, alcohol use and family history of upper gastrointestinal cancer. Significant associations were observed between FasL expression and male sex (r = 0.17), tobacco use (r = 0.17), and family history of UGI cancer (r = -0.18) (all p values < 0.01). Mild correlation was seen between caspase 8 expression and alcohol use, but it did not reach statistical significance. The Spearman correlation between the seven apoptosis biomarkers and four clinico-pathologic features were also studied (Table 4). Histological differentiation was strongly correlated with Fas, caspase 10, and bcl-2 expressions (r = -0.26 to -0.36, all p values < 0.0001); lower (but still statistically significant) correlations were observed with FADD, caspase 8, and FasL. In addition, significant associations were also seen for tumor grade with Fas, FADD, and caspase 10, and for tumor stage with Fas expression.

**Table 4:** Spearman correlations between 7 apoptosis biomarkers and 5 risk factors and 4 clinico-pathologic features^1^

	FasL	Fas	FADD	pFADD	Caspase8	Caspase10	Bcl2
**Risk factors**							
Male (yes)	0.17**	0.01	0.05	0.02	-0.02	0.03	0.00
Age (yrs)	-0.01	0.02	-0.06	-0.05	0.10	0.00	0.00
Tobacco use (yes)	0.17**	0.05	0.08	-0.07	0.00	0.10	0.02
Alcohol use (daily or weekly)	0.00	-0.02	0.20**	-0.09	0.13*	0.09	0.02
Family history UGI cancer (yes)	-0.18**	-0.15*	0.00	0.04	-0.05	0.02	-0.01
							
**Clinico-pathologic features**							
Tumor grade (I -- IV)	-0.10	-0.20**	-0.18**	0.08	-0.07	-0.19**	0.02
Tumor stage (1 -- 4)	0.00	-0.14*	-0.05	0.10	0.01	0.03	0.03
Lymph node metastasis (yes)	0.02	0.01	-0.04	0.06	0.00	0.01	0.02

Degree differentiation (poor)	-0.15*	-0.26***	-0.21**	0.03	-0.17**	-0.36***	-0.30***

^1 ^N varies from 235 to 253 and is less than 265 total because none of 7 biomarkers had data for all 265 ESCC cases

* p < 0.05

** p < 0.01

*** p < 0.0001

Relations to survival adjusted for these five risk factors and four clinico-pathologic features are shown in Table 5. Higher tumor stage [hazard ratio (HR), 1.92; 95% confidence interval (95% CI), 1.14-3.23] and presence of lymph node metastasis (HR, 2.17; 95% CI 1.59-2.95) were significantly and independently associated with death.

**Table 5:** Adjusted hazard ratios^a ^for death by risk factors and clinico-pathologic features in ESCC cases (N = 260)

Variable	Hazard Ratio	95% CI	*P*-value
Gender (male)	1.09	0.70 - 1.70	0.69
Age (years)	1.01	0.99 - 1.03	0.28
Tobacco use (yes)	1.08	0.70 - 1.67	0.72
Alcohol use (daily or weekly)	1.00	0.67 -- 1.47	0.98
Family history of UGI cancer (yes)	1.00	0.72 - 1.40	1.00
Tumor grade (I -- IV)	1.18	0.90 - 1.54	0.24
Tumor stage (1 -- 4)	1.92	1.14 - 3.23	0.01
Metastasis (yes)	2.17	1.59 - 2.95	< 0.0001

Degree differentiation (poor)	1.07	0.76 - 1.50	0.71

^a^Model includes all 9 variables shown (gender, age, tobacco use, alcohol use, family history of UGI cancer, tumor grade, tumor stage, metastasis, degree differentiation)

## Discussion

The results of correlation analyses confirmed that these biomarkers in the FasL-Fas apoptotic pathway were closely related. The expression of p-FADD was generally low in this study, and it may explain why we did not detect correlations between p-FADD and other markers. Bcl-2 inhibits BAX (Bcl-2-associated X protein)/BAK (Bcl-2-antagonist/killer1) proteins, which induce the permeabilization of the outer mitochondrial membrane, a crucial step for apoptotic cell death [7]. Caspase 8 is involved in functions related to bcl-2 and BAX/BAK proteins. Bcl-2 was not significantly correlated with caspase 8, but was mildly associated with caspase 10. Although caspase 10 does not seem to directly interact with bcl-2 in the apoptosis pathway, correlation of expression may be anticipated, given the complexity of apoptosis regulation.

In accord with our findings, Xue *et al. *also previously reported that Fas and FasL were not related to disease-free survival in ESCC [8]. In contrast, Kase *et al. *observed significantly longer ESCC-free survival in patients with Fas-positive (versus Fas-negative) tumors, and in FasL-negative (versus FasL-positive) tumors [9]. Shibakita *et al. *reported that Fas expression was an independent prognosticator for recurrence-free survival, but that FasL expression did not influence ESCC survival.[9] Studies of the prognostic significance of caspase 8 are limited to a single previous report in which no effect on survival was noted [10]. There are no published studies thus far on the prognostic significance of caspase 10 in ESCC.

FADD did not predict survival in ESCC in our study, a finding similar to that of Chang *et al *[11]. In contrast, Xue *et al. *reported that FADD expression correlated with decreased survival in ESCC [8]. Induction of p-FADD results in suppression of cancer cell growth and invasion through reduction in the non-phosphorylated form in prostate cancer [12]. Consistent with two previous reports, we also were unable to relate bcl-2 expression to prognosis [13,14]. Chang *et al. *found that bcl-2 expression correlated with better survival, and was an independent prognostic factor after multivariate analysis[11]. Similar findings were noted by Parenti *et al. *and Ohbu *et al.*, however, in both studies bcl-2 was not an independent prognostic value after adjustment for other variables in multivariate analysis [15,16].

FasL expression showed significant correlation with three risk factors including male sex, tobacco use, and family history of UGI cancer (Table 4). Fas had mild association with family history of UGI cancer. Interaction of Fas and FasL initiates Fas-mediated apoptosis and transmits signals to the downstream of the pathway. It suggests that these risk factors might closely relate to Fas-mediated apoptosis pathway and contribute to the pathogenesis of ESCC. Among the clinico-pathologic features we studied (Table 4), tumor grade was significantly associated with Fas, FADD and caspase 10. Histological differentiation of ESCC was significantly correlated with the target biomarkers except p-FADD, which exhibited very low expression. In the present study, we used a TMA to analyze a larger number of ESCC cases for apoptotic pathway markers than any previous report in the literature. Further, performance of IHC on a single slide under identical conditions should minimize variability in staining and thus enhance the reliability of our results. Despite the size and other favorable characteristics of the current study, the prevalence of protein positivity for the apoptosis pathway markers examined was low (≤ 15%) and the differences in survival between protein expression positive versus negative groups were small, resulting in only limited power (<10%) to distinguish the small differences in survival actually observed between groups here. For the markers with the highest positivity (ie, 15%) in this study, we had good (ie, 80%) power to detect only much larger hazard ratios that were observed here (ie, 3.4 or greater).

## Conclusion

While the current study evaluated only protein expression in relation to survival and not the potential use of these biomarkers in the early detection of ESCC, the generally low prevalence of expression positivity indicates that they would not be suitable candidates for early detection markers. We were unable to identify a role for biomarkers in the FasL-Fas apoptotic pathway and prognosis in ESCC.

## Competing interests

The authors declare that they have no competing interests.

## Authors' contributions

NH, PRT, and SMH designed the study. NH, JZS, and QHW were responsible for management of patients' data and tissues. MT and SMH performed IHC and provided the scoring data. CG, NH, and PRT performed statistical analysis. MT and SMH wrote the manuscript, and NH and PRT helped editing the manuscript. All authors read and approved the final version of the manuscript.

## Pre-publication history

The pre-publication history for this paper can be accessed here:

http://www.biomedcentral.com/1471-2407/9/310/prepub
